# The challenges of implementing hybrid baselines for the interpretation of longitudinal behavioral data from individuals

**DOI:** 10.1038/s41746-026-02668-5

**Published:** 2026-04-22

**Authors:** Sandra Anna Just, Enrico Tedeschi, Einar Holsbø, Karl Øyvind Mikalsen, Lars Ailo Bongo, Philipp Homan, Brita Elvevåg

**Affiliations:** 1 Department of Clinical Medicine, UiT—The Arctic University of Norway, Tromsø, Norway; 2 Department of Psychiatry and Neurosciences, Charité Campus Mitte, Charité—Universitätsmedizin, Berlin, Germany; 3 Department of Computer Science, UiT—The Arctic University of Norway, Tromsø, Norway; 4 Center for New Antibacterial Strategies (CANS), UiT—The Arctic University of Norway, Tromsø, Norway; 5 The Norwegian Center for Clinical Artificial Intelligence, University Hospital of North Norway, Tromsø, Norway; 6 Neuroscience Center Zurich, University of Zurich and ETH Zurich, Zurich, Switzerland; 7 Department of Adult Psychiatry and Psychotherapy, University of Zurich, Zurich, Switzerland

**Keywords:** Neuroscience, Psychology, Psychology

## Abstract

Establishing whether observed behavioral differences reflect meaningful change in an individual necessitates baselines specific to the individual and task. Automated hybrid solutions combine adaptive baselines with fixed thresholds. Applying this approach to behavioral science harbors challenges: the pronounced gap between observable measurement and underlying construct means ground truth is typically unavailable. A stepwise framework is proposed to determine and evaluate the validity of baselines for longitudinal behavioral measurements.

## Group-level norms are not suitable for the interpretation of longitudinal data from individuals

Behavioral science domains such as psychology have overwhelmingly been built around group-level inference, aiming to understand inter-individual differences. The field lacks a comparable tradition of treating intra-individual change as a primary object of interpretation. This gap is significant because determining how a person changes over time requires different conceptualizations and statistical approaches than determining how individuals differ from one another at a given time^1,2^. Group-level norms help quantify how a single, cross-sectional measurement compares to a reference population. However, these norms are not appropriate when evaluating intra-individual change in longitudinal behavioral measurements^3^. For example, when an individual’s memory performance is assessed repeatedly over time, group-level norms are useful to indicate how task performance compares to a group average at *each single measurement*. They are *not* useful for understanding how the individual’s measurements relate to each other over time, how the measurements are augmented – and potentially transformed – by practice or learning effects, or when they represent a change.

**Fig. 1:**
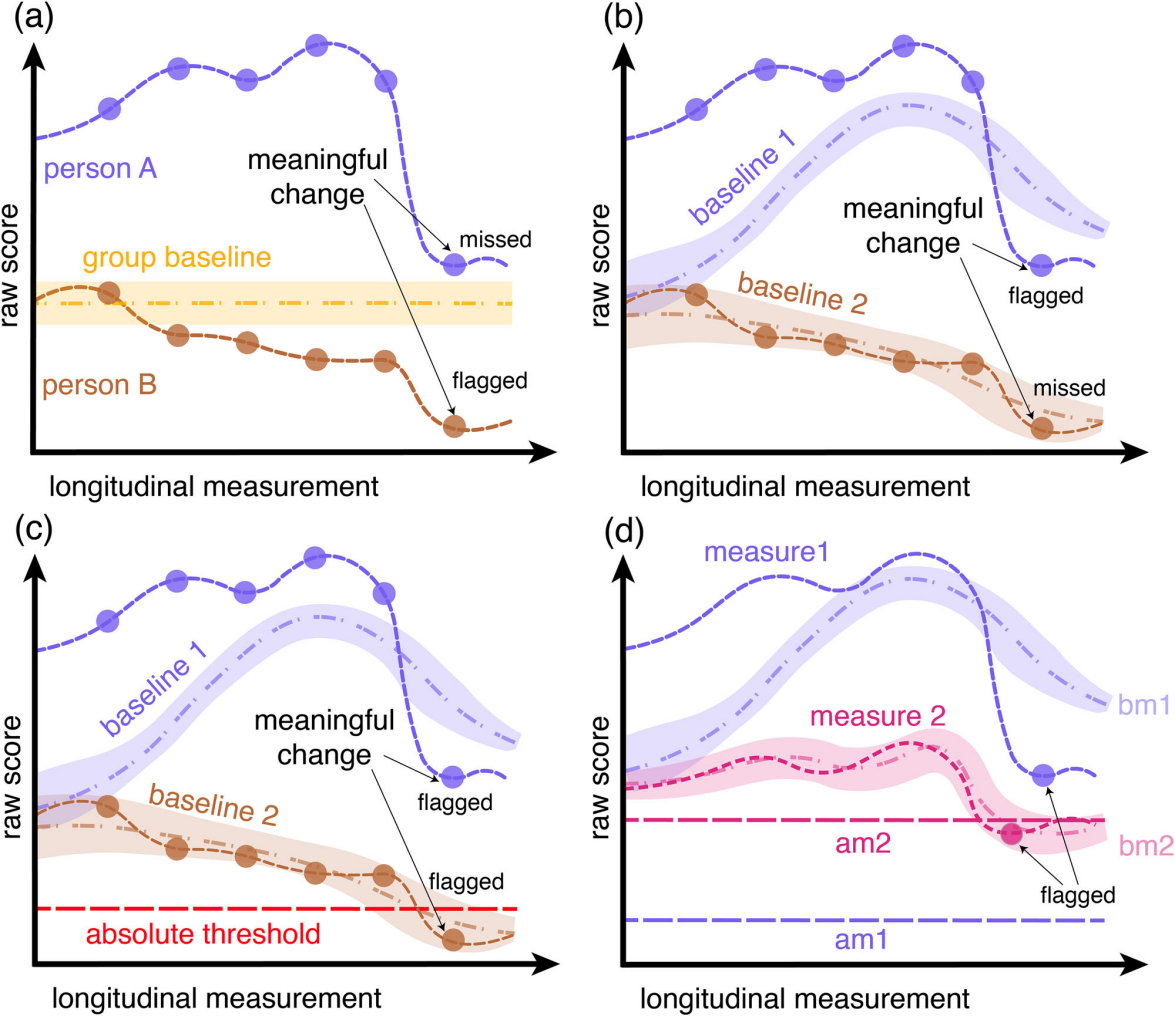
Hybrid baselines for the interpretation of longitudinal behavioral data. The four panels show hypothetical line charts of longitudinal measurements. (**a**–**c**) show the trajectory of raw scores from two individuals for the same behavioral measure, while (**d**) shows the trajectory of raw scores from one person in two different measures. (**a**) Group-average baselines ignore different individual averages and trajectories. Scores from person A (purple) and person B (brown) are plotted alongside a group baseline that represents the group’s average (yellow) with a defined uncertainty range (shaded light yellow area). Person A shows an individual average of scores far above the group baseline. Based on that baseline, the sudden decline in their last measurement is missed as it is still considered ‘above average’. In comparison, scores from person B decline more gradually but are flagged as they lie below the group-average baseline. (**b**) Individual baselines support interpretation of longitudinal data. Plotted scores are identical to (**a**), but now each person has an individual baseline (baseline 1 and 2). While both baselines start off at the group average, they continuously adapt to the person’s measurements. In contrast to (**a**), person A’s last measurement is now flagged as it falls below their adapted individual-average baseline. Person B’s baseline adapts to the gradually declining scores so that their decline is now missed. (**c**) Individual baselines need to be combined with absolute thresholds. Plotted scores are identical to (**a**) and (**b**) but baselines are now combined with a fixed absolute threshold, defining which values should be flagged for all individuals for the given measure. This approach addresses the challenge of the gradual decline of scores in person B: Once their score falls below the absolute threshold, it is flagged. (**d**) Individual baselines need to be task-specific. Scores from one person are plotted for measure 1 (purple) and measure 2 (magenta) alongside task-specific baselines (bm1, bm2) and absolute thresholds for measure 1 and 2 (am1, am2). This is to show that individual averages and trajectories differ between measures and require task-specific computation.

Intra-individual measurements taken over time need to be interpreted relative to a baseline. In behavioral science, group averages are unsuitable as individual baselines. Treating a group average as an individual baseline assumes ergodicity, whereby individual-level statistical properties equal those observed at the group level. This assumption likely does not hold for most behavioral processes^4^. As shown in Fig. 1 (a), the use of group averages as baselines conflates variability *between* individuals with variability *within* an individual and mischaracterizes intra-individual differences and putative change^3^. Moreover, static group-level norms ignore temporal variability, which is essential for distinguishing routine fluctuations from unusual patterns in a time series^5^. Reliable interpretation of longitudinal behavioral data, therefore, requires baselines derived from an individual’s prior measurements rather than group averages^6^. This principle applies broadly across behavioral research, including clinical trials. For example, although psychiatric assessment is often criterion-based (i.e., is a symptom present or not?), changes in symptom severity must still be interpreted relative to an individual’s baseline psychopathology. To illustrate, consider a patient who experiences auditory hallucinations multiple times per day, who will have a fundamentally different baseline than one who hears voices only a few times per month. A change to experiencing hallucinations once a week would represent improvement for the former but worsening for the latter. Individual baselines are therefore essential for accurate interpretation of symptom trajectories in clinical research.

## The necessity for data-driven individual baselines in behavioral science

Longitudinal behavioral data can now be collected at an unprecedented density and frequency – through remote, digitized, or automated assessments^7–10^. These measurements produce time-series datasets, whose value lies in the trajectories and dependencies they reveal rather than single measurements. Moreover, as one task alone is rarely used to infer human behavior, measurements are collected across different assays. Such repeated and combined observations contain rich information about intra-individual patterns and dependencies, but also present methodological challenges that cannot be addressed using group-level norms. The challenge is that the sheer volume and density of repeated measurements transform the very nature of the data being collected. There are two reasons for this. First, the experimental process becomes more familiar to the participant over time, and they learn what is expected from them and may perform better. Indeed, most cognitive and behavioral tasks are associated with familiarity, learning, and practice effects. Second, as the years, months, weeks of assessment go by, inevitably, participants will be required to engage in behavioral tasks where stimuli material may overlap with tasks they have previously taken part in. The observed effects will be the result of a combination of effects (e.g., multiplicative and task transfer effects). Thus, as data collection continues, the datasets have the potential to get messier and more complex (in terms of discerning what is attributable specifically to the task construct versus other variables).

Clinicians can integrate their patients’ individual history and trajectories during evaluation and clinical encounters. However, when automated systems are to interpret longitudinal behavioral data, they require explicit computational rules for this integration. The complexity and volume of remotely collected time-series data make it impractical to establish baselines by manual inspection alone. Therefore, computational methods are absolutely essential to systematically derive individual baselines from the vast– and growing– set of time-series data. By statistically characterizing an individual’s relative patterns directly from their measurement history, these methods do not rely on group averages and can adapt baselines continuously as individual trajectories evolve over time. This approach acknowledges that individual baselines are not static but may gradually change, for example, because of learning or aging. Individual baselines may also mitigate the issue that manifest measurements in behavioral science are not equal to the underlying construct that they intend to measure. A given measurement (e.g., a score in a memory test) represents only an approximation of the latent construct (e.g., memory), containing systematic variation and noise, which are both unwanted and unknown. By aggregating information across multiple timepoints, individual baselines may better approximate an individual’s true expression in the construct, provided that the measures are carefully designed to appropriately assay the construct and that data quality is adequate. This approach does not depend on establishing ground truth with additional measures (e.g., clinical ratings) that would introduce measurement errors on their own.

## Computational hybrid baselines for the interpretation of longitudinal behavioral measurements

The computation of hybrid baselines can be automated. The purpose is to support the interpretation of longitudinal behavioral measurements from individuals. As illustrated in Fig. 1 (b-d), hybrid baselines combine task-specific, adaptive, individual baselines and task-specific absolute thresholds.

Individual baselines are useful because they can continuously adapt to an individual’s prior measurements for a given task, capturing their individual average, variability, and trajectory more meaningfully than static group-level norms and in a manner that is more relevant to the context. Computing an individual baseline requires accounting for the individual’s developing trends (long- and short-term), and variability over time. As an example, the baseline at timepoint $t$ could be expressed as a weighted, moving average over a sliding window of the $w$ most recent measurements:

1 $${b}_{t}=\frac{{\sum }_{i=1}^{w}{w}_{i}{x}_{t-1}}{{\sum }_{i=1}^{w}{w}_{i}}$$

where ${x}_{t-1}$ is the measurement $i$ steps before timepoint $t$, $w$ is the window size (the number of measurement time points included), and ${w}_{i}$ is the weight assigned to measurement $i$. Recency weighting assigns greater weight to more recent measurements, allowing the baseline to be more responsive to recent change–if deemed suitable for the construct of interest. Equal weights (${w}_{i}=1\,$ for all $i$) yield a simple moving average. To reduce the influence of outlier measurements, a trimmed mean may be calculated by excluding very high and low values when computing the moving average.

The uncertainty range around the baseline (visually represented as the shaded area surrounding baselines in Fig. 1) defines the range of values considered ‘average’ for an individual on a specific task and therefore not indicative of meaningful change. A simple way of defining such a range is to say that staying within some number of standard deviations of the baseline is average:

2 $${b}_{t}\pm k\cdot \max ({\sigma }_{t},{\sigma }_{min})$$

where ${b}_{t}$ is the baseline estimate at time $t$, ${\sigma }_{t}$ is its standard deviation, $k$ is a scaling factor controlling the width of the range. Taking the baseline as a weighted average, a natural standard deviation estimate would be the corresponding weighted standard deviation. This would update continuously to reflect the certainty or confidence of the calculated baseline: it is wider when there are only a few measurements, missing values, or high fluctuation in an individual’s performance on a particular task, and narrower when many consistent measurements are available.

It should be noted that, of course, it is not always the mean that is interesting but rather the variance, and the pattern of variance. Indeed, for many simple tasks (e.g., response time data), the variance is often more interesting and useful, although for more complicated tasks (e.g., complex decision tasks), the mean is usually sufficient.

An adaptive baseline alone is insufficient for interpreting longitudinal behavioral data: without absolute thresholds, an adaptive baseline can drift, gradually accommodating slow but clinically meaningful change that will be missed^11^. This is problematic in psychiatric settings, where patients with diagnoses such as schizophrenia, bipolar or depressive disorder can show early warning signs of a relapse, gradually deteriorating over weeks^12–15^. Interpretation solely based on an adaptive baseline could miss this gradual change. Therefore, for each measure or assay, researchers need to define which measurement values should be flagged across all individuals, independent of their individual average. As shown in Fig. 1 (c), this approach prevents gradual but potentially meaningful change from being missed. Absolute thresholds also accommodate criterion-based measures, where certain values indicate clinical concern regardless of individual history or prior change. For example, an entire night without sleep warrants clinical attention in patients with conditions such as bipolar disorder, even in chronically poor sleepers. These absolute thresholds can be based on group-level statistics, expert knowledge, and/or task-specific considerations. The observation of a measurement score triggers an alert if it exceeds either criterion: falling outside the uncertainty range of the individual’s baseline or crossing an absolute threshold, whichever occurs first. In high-risk applications, such as those informing clinical decision-making, more sensitive thresholds may be chosen to prevent missing clinically relevant change. Each new behavioral measurement can be automatically compared against both the individual baseline and the absolute threshold. This approach offers a scalable, adaptive, and reliable interpretation of longitudinal behavioral measurements.

However, one size does not fit all in behavioral measurements. Therefore, when selecting the parameters and their values for the hybrid baseline approach, decisions need to be specific to the (i) task, (ii) individual, and (iii) context and be guided by (iv) theoretical assumptions about the latent construct that is intended to be assayed.

## The challenges for data-driven individual baselines in behavioral science

The statistical assumptions underlying the hybrid baseline approach are not unique to behavioral science but widely used in other domains that analyze time-series data, such as computer science, forecasting science, and physiology^16–18^. The distinct contribution of the present approach lies not in the mathematical operationalization of baselines itself, but in its conceptualization for the specific challenges of longitudinal behavioral data from individuals, which serves as an indirect proxy for the cognitive or mental construct of interest. The challenges arise from the gap between observable behavioral measurements and latent theoretical constructs and concern (i) the lack of ground truth, (ii) the inherent imprecision of latent constructs, and (iii) measurement errors. These challenges are well-known in behavioral/measurement science, but are amplified to an unprecedented and unknown level when measuring behavior across time and can interact and reinforce one another.

The first challenge is the *lack of ground truth* in behavioral science that exists because of the dependence upon constructs that are merely inferences regarding the unobservable yet assumed cognitive processes and mental events. This gap between observable measurement and underlying construct exists in other disciplines also (e.g., a heart rate does not directly map to metabolic health), but it is considerably more pronounced in behavioral science, where constructs such as memory or happiness generally lack a direct mapping to underlying physiology and, as such, seem relatively unconstrained. Since the ground truth of these constructs cannot be determined conclusively, and approximations of ground truths by experts are rarely available a priori, an approach to baselines in behavioral science cannot be evaluated for its *accuracy* in terms of detecting a discrete event. Rather, the focus needs to be on *validity,* and the issue is better framed as one of *prediction* rather than signal detection. Validity in this context refers to how well the baseline model estimates the probability that, given an individual’s measurement history, an observed change reflects a meaningful difference in how the construct is expressed at the present timepoint relative to previous observations.

The lack of ground truth is closely related to a second challenge: the inherent *imprecision* in defining the latent constructs in behavioral science. In domains such as physiology, these definitions are more precise. For example, although heart rate per minute may also contain measurement noise, it corresponds closely to the cardiac activity it is intended to measure. The temporal pattern of the cardiac activity is well-defined and corresponds directly to the temporal resolution of heart rate as a measure. This is *not* the case for cognitive, mental, and emotional constructs whose definitions are inherently imprecise. This imprecision is aggravated in longitudinal designs, which must specify not only the characteristics and boundaries of the construct but also its temporal structure—that is, the expected stability and the rate at which meaningful change occurs.

While measurements will always include some level of noise, the third challenge is that longitudinal behavioral measurements are particularly susceptible to *systematic errors*. The very process of collecting behavioral measurements frequently changes the signal value, its potency, and validity. Repeated cognitive testing, for example, can produce learning or practice effects—specific to the individual and task. Unlike random noise, which baseline models can accommodate statistically, the very act of repeated measurements systematically augments, distorts, and transforms the signal, thereby undermining the validity of baselines over time. Baseline models with higher validity will be those that more accurately partition observed variance into variance attributable to the construct of interest and variance attributable to measurement error.

## How to determine hybrid baseline parameter values

When annotated datasets with ground-truth labels are available, selection and evaluation of parameters for the baseline approach can follow a standard supervised framework: Models are trained on data from one timeframe, tuned on a second, and tested on a third timeframe. Different parameter configurations are compared against simpler baseline approaches (e.g., fixed baselines, group averages) and against each other. Performance evaluation focuses on the detection of ground truth events, i.e., determining the baselines’ accuracy through metrics such as sensitivity and specificity.

**Fig. 2:**
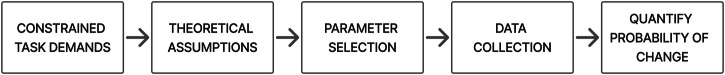
A stepwise approach to quantifying the probability of meaningful change in longitudinal behavioral observations from individuals.

However, in most cases in behavioral science, ground truth is not available or determinable. This warrants a carefully planned procedure for determining the parameters of the given baseline model. No doubt there are several possible approaches, but we illustrate here one possible stepwise approach (see Fig. 2):

- Start simply with constrained task demands
  Careful design of the actual task that will be used to elicit the construct ensures that many variables are controlled for, even before getting to the stage of thinking about how to model the baselines. Consider a basic reaction time task where individuals are to press as quickly as possible a lever in response to a series of signals. The response times are used to make inferences about the processes underlying the response. Reaction time speeds are generally slower if there are more variables (e.g., several possible signals to respond to), and thus the underlying construct is regarded to be more complex. In contrast, faster response speeds indicate that the task is easier, likely because of fewer underlying cognitive steps in the construct^19^. Such a design ensures that the task demands are constrained (e.g., of possible signals or types of responses) and that they can be systematically varied so as to understand better the underlying constructs. Numerous metrics can be derived that may be useful as inputs for computing a baseline; reaction times or inter-response rates could be used to examine how response patterns may change within the testing window. For some application purposes, an average may of course be useful, but in other contexts the variance and changes in this are necessary to capture the dynamics of behavior^20^ and volatility of mood, for example^21^. Indeed, signals may have very different temporal, spatial, and spectral resolutions, and their value will be different depending upon their application context^22^.
- Formulate theoretical assumptions
  The application context of the construct of interest is crucial, contributing to its definition and, crucially the importance of the scale of its temporal structure. Assumptions about the temporal structure of a construct concern how stationary it is, and in what timeframe potential meaningful change is likely to occur. This determines what timeframe is valid for deriving an individual’s baseline (e.g., the window size of measurement points selected for baseline computation) and what the time scale is for eventual baseline adaptations (e.g., a week, month, or year). Additionally, assumptions need to be made about potential sources of systematic error, for example, whether learning or practice effects are expected, as these effects systematically change an individual’s measurement values over time, which the baseline model must account for. These theoretical assumptions need to be articulated before any model specifications are made.
- Determine parameters
  Parameter selection is driven by the theoretical assumptions about the construct of interest as well as aspects of the task, individual, and context, which is why parameters cannot be selected on purely statistical grounds. In the case of the aforementioned reaction time task, if gaining a basic understanding of cognition is the purpose of data modeling, theoretical assumptions need to be made about cognition and its temporal structure. As cognitive constructs are generally considered to be fairly stable, a larger timeframe/window size (months to years) in which measurements are weighed equally seems a suitable parameter for determining an individual’s baseline in for example a tapping task. In contrast, mental states and emotions are regarded as more changeable. If the construct of interest is mood, it is logical to choose shorter window sizes (on the order of weeks or days) and to assign a higher weight to more recent measurements, for example, for a simple mood questionnaire. When applying the model to a specific individual and context—for example to measure mood volatility in a patient with a high risk of mood swings that has a particularly severe negative affective component and an associated risk of engaging in self-harm behavior—then the window size and adaptation rate need to reflect the speed at which clinically relevant mood changes are expected to unfold in that individual in this context for that task.
- Collect enough data points
  In the absence of ground truths with most constructs, the best approximation of an individual’s baseline in the latent construct of interest can be compiled from their measurement history. The validity of that baseline will depend on the number of available observations. Consider that in traditional norming studies in psychology, such as measuring intelligence, where the group norm was essential to understand in terms of calibrating task difficulty, data would have been collected from a sample on the scale of ten thousand people or more, for a test with thousands of individuals populating the various age bands to compute this normative data. Drawing from this, a norming approach for an *individual—*that will be used to compare that individual’s performance *relative* to themselves—that aims for high validity, sampling on the scale of at least a hundred data points, ideally thousands, seems wise. Collecting that many data points for an individual to determine a valid baseline will often be unrealistic. An alternative can be to—in addition—consider the measurement history from that same individual for other variables of a similar type of the same construct. If deemed necessary, in some circumstances, it if can be established somehow that certain individuals are sufficiently similar on crucial variables, then one may consider including measurement histories for the same variable in these similar individuals by partial pooling of measurements.
- Quantify probability of change
  Framing baselines as signal detection models is not useful in behavioral science. Instead, we propose conceptualizing baselines for measuring intra-individual change over time as forecasting models^18^ with a certain validity, not accuracy, in quantifying the (im)probability of change in a nuanced fashion.
  An individual’s baseline is based on their measurement history that could be considered as a stochastic process ${Y}_{1},{Y}_{2},\ldots {Y}_{n}$, where ${Y}_{i}$ is a random variable representing an observation at time point $i$. A function $f$ estimates the probability of the individual’s next observation in the time-series:
  3 $$P\left({Y}_{n+1}\right)\sim f\left(\theta ,{Y}_{1},{Y}_{2},\ldots ,{Y}_{n}\right),$$
  where $\theta$ represents aspects specific to the task, individual and context as well as theoretical assumptions about the latent construct (notably measurement error, correlation between consecutive measurements, distribution of parameters).
  Given baseline observations ${Y}_{i}={y}_{i},\ldots ,{Y}_{n}={y}_{n}$ and having decided on a model $f$, the next observation ${Y}_{n+1}={y}_{n+1}$ can be evaluated against the forecasting distribution: how likely is a deviation of at least this magnitude in this model? A low probability indicates that this observation differs meaningfully from previous ones. The model is thus not evaluated for accuracy, but for its validity in determining the probability of an individual’s behavioral patterns over time. This evaluation always needs to be specific to the task, individual and context.

## Conclusion

Baseline models are needed for the meaningful interpretation of longitudinal behavioral data. In the case of clinical applications, there is a fundamental requirement that appropriate baselines are both useful and safe. However, the inherent challenges in measuring human behavior are magnified to an unprecedented degree in longitudinal designs, making it practically impossible to directly apply baseline models from other disciplines. Different conceptualizations of baselines were presented, from group averages to adaptive individual baselines derived from measurement history and hybrid models combining individual baselines with absolute thresholds. Finally, a framework was proposed that advocates starting simple, conceptualizes individual baselines in behavioral science as forecasting models and in doing so embraces—rather than eliminates—the complexity and imprecision that is intrinsic to human behavior.

## Data Availability

No datasets were generated or analysed during the current study.
